# Spore Powder of *Paecilomyces hepiali* Shapes Gut Microbiota to Relieve Exercise-Induced Fatigue in Mice

**DOI:** 10.3390/nu14142973

**Published:** 2022-07-20

**Authors:** Tianyue Guan, Shuoshuo Li, Qijie Guan, Jin-Song Shi, Zhen-Ming Lu, Zheng-Hong Xu, Yan Geng

**Affiliations:** 1 School of Life Sciences and Health Engineering, Jiangnan University, Wuxi 214122, China; guantianyue02@163.com (T.G.); li_shuoshuo0714@163.com (S.L.); shijs@163.com (J.-S.S.); 2 National Engineering Laboratory for Ce Real Fermentation Technology, Jiangnan University, Wuxi 214122, China; qijie.guan@jiangnan.edu.cn (Q.G.); zmlu@jiangnan.edu.cn (Z.-M.L.); zhenghxu@jiangnan.edu.cn (Z.-H.X.); 3 Jiangsu Engineering Research Center for Bioactive Products Processing Technology, Jiangnan University, Wuxi 214122, China; 4 The Key Laboratory of Industrial Biotechnology, Ministry of Education, School of Biotechnology, Jiangnan University, Wuxi 214122, China

**Keywords:** gut microbiota, *Paecilomyces hepiali*, anti-fatigue, spore, functional food

## Abstract

*Paecilomyces hepiali*, a fungal strain isolated from natural *Ophiocordyceps sinensis*, contains similar pharmacologically active components, has been used widely as a substitute of *O. sinensis* in functional food and medicine. However, the components and anti-fatigue effects of *P.hepiali* spores and their mechanisms of action are largely unknown. Here, we compared the chemical composition in *P.hepiali* spore (HPS) and mycelium (HPM) by liquid chromatography with tandem mass spectrometry analysis. We found 85 metabolites with significant differences, and HPS contains more L-Malic acid, Oxalacetic acid, Fructose-1,6-bisphosphate, and L-Arginine than HPM. Then we evaluated their anti-fatigue effects and regulatory effects on the gut microbiota in mice. The forced swimming time (SW) was only significantly increased in HPS groups: the high and low dose of the HPS group was 101% and 72% longer than the control group, respectively. Both HPS and HPM treatment decreased lactic acid, blood urea nitrogen, creatine kinase while increased lactate dehydrogenase (LDH) levels in the blood. Moreover, mice treated with HPS and HPM showed less skeletal muscle fiber spacing and breakage. The relative abundance of *Alistips*, *Eubacterium*, *Bacterium*, *Parasutterella*, and *Olsenella* in the gut microbiota of the HPS group was higher than that in the HPM group through 16S rRNA gene sequencing analysis. These changes may be related to the regulation of nucleotide, amino acid, and carbohydrate metabolism. Correlation analysis between the gut microbiota and fatigue-related indicators suggested that *Alistips*, *Clostridium*, *Akkermansia*, *Olsenella*, and *Lactobacillus* were positively correlated with the SW and LDH content. Our findings demonstrated that HPS has beneficial anti-fatigue effects by regulating gut microbiota.

## 1. Introduction

Fatigue is a kind of sub-health physiological state with various causes, which can be divided into mental fatigue and physical fatigue [1,2]. The cause of fatigue is believed to be the imbalance of metabolism, the lack of energy supply substances and the inhibitory effect of the central nervous system [3]. Excessive exercise will lead to excessive accumulation of metabolites such as lactic acid and urea nitrogen in humans and animals [4,5]. Once these metabolites in the body cannot be reduced through rest, it will cause changes in the internal environment and produce fatigue manifestations such as pH fluctuations and muscle soreness [3]. In the state of fatigue, the human body’s response ability becomes slow, flexibility and coordination are reduced, resulting in a decline in work efficiency, and reducing the body’s immunity, which ultimately leads to the occurrence of diseases [6]. Therefore, research on the treatment and prevention of fatigue has important value [7]. 

Existing studies have shown that the anti-fatigue ability of substances is related to the regulation of gut microbiota [8,9]. The increase in the abundance of probiotics in the intestine can reduce the body’s fatigue and enhance sports functions. Gut microbiota is the most complex micro-ecosystem possessed by the human body and plays a vital role in regulating the host’s biochemical and physiological state [10]. Dietary regulation is a crucial way to modulate the structure and activity of the gut microbiota, which in turn can impact host physiology [11]. Many Chinese herbal medicines can significantly promote the growth and activity of probiotics on the gut microbiota [12].

The caterpillar fungus *Ophiocordyceps sinensis* (syn. *Cordyceps sinensis*) is one of the world’s most valuable biological commodities. Due to low reproduction, great market demand and climate change, *O.sinensi* has been overharvested [13]. *Paecilomyces hepiali*, a fungus isolated from natural *O.sinensis*, is an important alternative to *O.sinensi* in medicine and health foods market [14]. The mycelia of this fungus are cultivated by liquid-state fermentation, which contain similar bioactive components to *O.sinensi* such as alkaloids, cyclic dipeptides, steroids, organic acids, and polysaccharides [15,16,17]. *P.hepiali* can form spores in the liquid fermentation, and the asexual spores-based inoculation and recycling fermentation method notably reduces cost and labor, and improve the production efficiency and controllability of the fermentation process [18]. The fermented mycelia have demonstrated a series of pharmacological activities in past studies, such as anti-oxidation, anti-fatigue, anti-cancer, and immune regulation effects [19,20]. However, the main components and the function of its spore are largely unknown. Here, we investigated the chemical composition in the fermented mycelia and spores of *P.hepiali*, and explored their anti-fatigue effects by an exhaustive swimming test in mice. Moreover, we studied their impact on gut microbiota. The result suggests that fermented spores of *P.hepiali* may be powerful modulators of the gut microbiota and may serve as functional foods.

## 2. Materials and Methods

### 2.1. Preparation of Mycelium and Spores of Paecilomyces hepiali

Paecilomyces *hepiali* 14065 obtained from China Center of Industrial Culture Collection (CICC) was cultured as described [18,21]. The fermented mycelia and spores of *P.hepiali* were collected as described [22].

### 2.2. Metabolite Extraction and Liquid Chromatography with Tandem Mass Spectrometry (LC-MS/MS) Analysis

Samples (60 mg) of spores and mycelia of *Paecilomyces hepiali* were dried with SpeedVac (Thermo Fisher Scientific, Shanghai, China). Each dried sample was resuspended in 500 µL methanol (−20 °C) centrifuged at 8000× *g* for 10 min. After centrifugation, the supernatant was collected and dried with SpeedVac. Before LC-MS/MS analysis, samples were dissolved with 150 µL 2-chlorobenzalanine (4 ppm) in 80% methanol and were filtered through the 0.22 µm membrane. A Vanquish liquid chromatograph connected to a Q Exactive Focus mass spectrometer (Thermo Scientific, Bremem, Germany) was used for untargeted metabolite analysis. The analytical column was an ACQUITY UPLC HSS T3 (150 × 2.1 mm, 1.8 µm, Waters) column maintained at 40 °C. The mobile phases in positive mode (ESI+) were 0.1% formic acid in acetonitrile/water (5/95, *v*/*v*, phase A) and 0.1% formic acid in acetonitrile/water (95/5, *v*/*v*, phase B), in negative mode (ESI-) were 0.1% 5 mM ammonium formate in water/acetonitrile (5/95, *v*/*v*, phase A) and 0.1% 5 mM ammonium formate in water/acetonitrile (95/5, *v*/*v*, phase B). The LC gradient profile was as follows (min/% of mobile phase B): 0.0/2, 1.0/2, 9.0/50, 12.0/98, 13.5/98, 14.0/2, and 20.0/2. The total runtime was 20 min. The flow rate was 0.25 mL/min, the column temperature was 40 °C, and the autosampler temperature was set at 8 °C. The ESI-MSn experiments were used with the spray voltage of 3.5 kV and −2.5 kV in positive and negative modes. The mass range was 81–1000 m/z with a resolution of 70,000. Data dependent acquisition (DDA) were performed with HCD scan. The normalized collision energy was set to be 30 eV, and dynamic exclusion was implemented for mass calibration.

### 2.3. Animals and Treatment

All mice procedures and protocols were approved by the Institutional Animal Care and Use Committee of Jiangnan University, Wuxi, China (Approval No. 20200430i0600710 (53)). Male C57BL/6 mice were obtained from Shanghai SLAC Laboratory Animal Co., Ltd. and kept in a specific pathogen-free (SPF) environment under standard laboratory conditions (24−25 °C, 12 h light/dark cycle). Five mice were housed in one cage with ad libitum access to distilled water and food. After acclimatizing to the barrier environment for one week, the mice were randomly and equally divided into six groups according to their body weight (*n* = 10 each group). The CTRL group was given AIN-93G purified diet. The other five group were given AIN-93G plus 0.375% Taurine, 0.675% *P.hepiali* mycelium (HPM), 0.225% HPM, 0.675% *P.hepiali* spore (HPS), and 0.225% HPS, respectively. All diets were prepared by Jiangsu-Xietong, Inc. (Nanjing, China) and kept the same number of calories. 

The forced swimming test was carried out at day 28 as described previously, with slight modifications [23]. A weight was attached to the tail of the mouse (7% mouse body weight), and then the swimming experiment was performed in a 55×40×40 cm water tank at a water temperature of 25 °C. The exhaustive swimming time of each mouse was recorded. After the final treatment, the mice were given two days of recovery and fasted at night of the 29th day. On the 30th day, the mice were forced to swim for 50 min without load and then rested for 30 min. Then the mice were anesthetized and sacrificed.

### 2.4. Sample Collection and Biomarker Determination

Blood was obtained from the posterior orbital veins. Serum was separated from the collected blood by centrifugation at 1500× *g*, 4 °C for 10 min. The mouse heart, heart, spleen, lung, kidney and liver were weighed and quickly placed in liquid nitrogen. These collected samples were stored at −80 °C for further analysis. The detection kits are used to detect the contents of lactic acid (LA), blood urea nitrogen (BUN), creatine kinase (CK), lactate dehydrogenase (LDH), catalase (CAT), glutathione peroxidase (GSH), superoxide dismutase (SOD) and malondialdehyde (MDA) according to the manufacturer’s instructions (Nanjing Jiancheng Bioengineering Research Institute, Nanjing, China).

### 2.5. Histopathological Examination

Mouse gastrocnemius muscle was fixed in 10% neutral-buffered formalin and then embedded in paraffin. The tissue section was subsequently stained with hematoxylin and eosin (H&E) and examined by microscopy (Nikon Corporation, Tokyo, Japan).

### 2.6. Gut Microbiota Analysis by 16S rRNA Gene Sequencing

Bacterial DNA was extracted from the collected stool samples, and 16S rRNA gene sequencing and analysis were completed by BGI (Beijing Genomics institution, Wuhan, China). Briefly, after filtering the original data, DADA2 denoised algorithm was used to get the amplicon sequence variant (ASV) table. The rank curve and species accumulation curve were generated by the ASV table. All the sequences were classified by Ribosomal Database Project (RDP) to get the taxa. The BGI online platform is used for species composition analysis, diversity analysis, and difference analysis. The microbiota datasets generated during and analyzed during the current study are available in the National Microbiology Data Center (NMDC, https://nmdc.cn, accessed on 7 December 2021), Biological Project No.: nmdc10017933.

### 2.7. Statistical Analysis

All data were expressed as the mean ± SD. Differences between the two groups were analyzed by one-way analysis of variance (ANOVA) with a post hoc Tukey’s test. *p* values <0.05 were considered significant.

## 3. Results

### 3.1. Characteristics of Spores and Mycelia of Paecilomyces hepiali

First of all, we analyzed the general composition of HPM and HPS. Both HPM and HPS contain high levels of protein, fat, and carbohydrate. Compared with the HPM, there were significant differences in the contents of ash, crude fat, coarse fiber, and total protein in HPS (Supplementary Figure S1). Then we found that 85 metabolites were significantly different in HPS and HPM by LC-MS/MS analysis (Supplementary Figure S2). The main categories are amino acids, purines, pyrimidine, carbohydrates, and their derivatives. The relative abundances of carbohydrates in HPS increased, such as Fructose-1,6-bisphosphate and D-Glucuronic acid, which provide energy for body movements. The relative abundances of malic acid and oxalacetic acid also increased in HPS, which are important substances in the process of energy metabolism. These results indicate that the compound composition of HPS may provide more energy to enhance the anti-fatigue ability of the body.

### 3.2. The Anti-Fatigue Effects of the Spore and Mycelia of Paecilomyces hepiali

**Figure 1:**
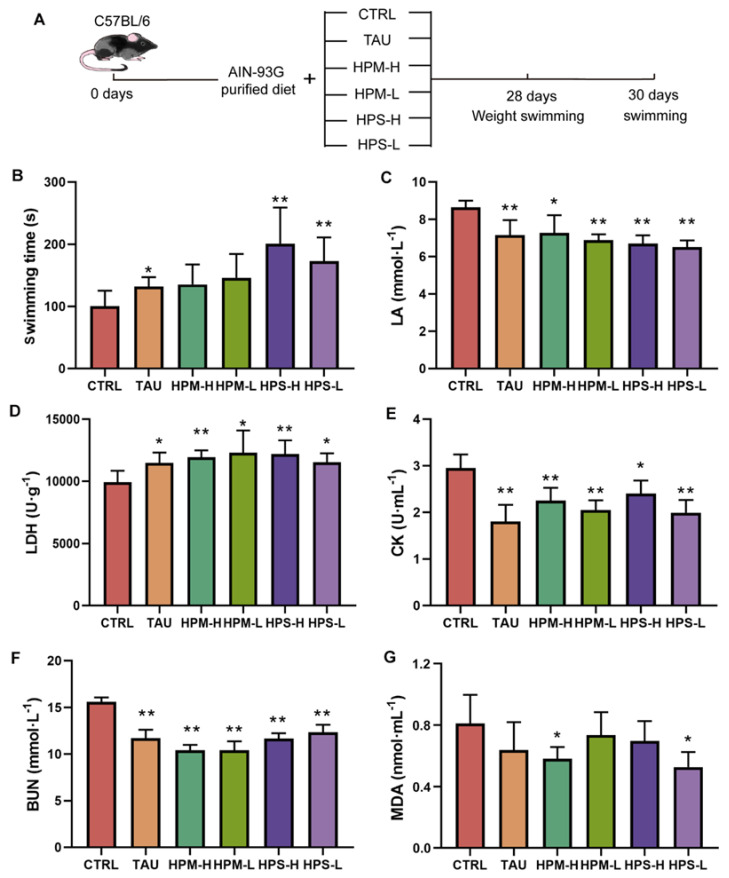
The anti-fatigue effect of the spore and mycelia of *Paecilomyces hepiali* on mice in each group. (**A**) Study design and diagram. The C57BL/6 mice were divided randomly into six groups. The CTRL group was fed the purified diet (AIN-93G), and the other five groups were fed with AIN-93G plus 0.375% Taurine (TAU group), 0.675% *P.hepiali* mycelium (HPM-H group), 0.225% HPM (HPM-L group), 0.675% *P.hepiali* spore (HPS-H group), 0.225% HPS (HPS-L group), respectively. (**B**) Swimming time; (**C**) lactic acid (LA); (**D**) Lactate dehydrogenase (LDH); (**E**) Creatine kinase (CK); (**F**) Blood urea nitrogen (BUN); (**G**) Malondialdehyde (MDA). Data are shown as mean ± SD. Asterisk (*) represents *p* < 0.05; Asterisk (**) represents *p* < 0.01.

To evaluate the anti-fatigue effect of HPS and HPM in vivo, we used a mouse model of exhaustive swimming-induced fatigue (Figure 1A). No significant treatment-related effects were observed with respect to body weight and the organ indexes of the heart, liver, spleen, lungs, and others (Supplementary Figure S3 and Supplementary Table S1). As expected, compared with the control group, the positive control TAU group, which was fed the anti-fatigue compound Taurine, can significantly extend the forced swimming time of mice. The high and low doses of HPM increased the forced swimming time, whereas it did not reach statistical significance (Figure 1B). The forced swimming time in HPS was significantly longer (*p* < 0.05), with HPS-H and HPS-L extending the time by 101% and by 72%, respectively (Figure 1B). These data indicate that HPS may have a better anti-fatigue effect than HPM in exhaustive swimming tests in mice.

### 3.3. Effects of the Spore and Mycelia of Paecilomyces hepiali on Blood Biochemical Indicators and Muscle Histopathology

Compared with the CTRL group, the lactic acid (LA) content in the TAU group, as well as HPM and HPS groups, was significantly decreased (Figure 1C). Meanwhile, compared with the CTRL group, the serum content of lactate dehydrogenase (LDH) was markedly increased in all the treatment groups (Figure 1D). The serum creatine kinase (CK) activity of the HPM-H, HPM-L, HPS-H, and HPS-L groups was significantly lower than the CTRL group, by 24%, 31%, 19%, and 33%, respectively (Figure 1E). There was more than 20% decreased Blood urea nitrogen (BUN) content in all the treatment groups (Figure 1F). Interestingly, the BUN was more significantly reduced in the HPM groups compared with the HPS groups (Figure 1F).

**Figure 2:**
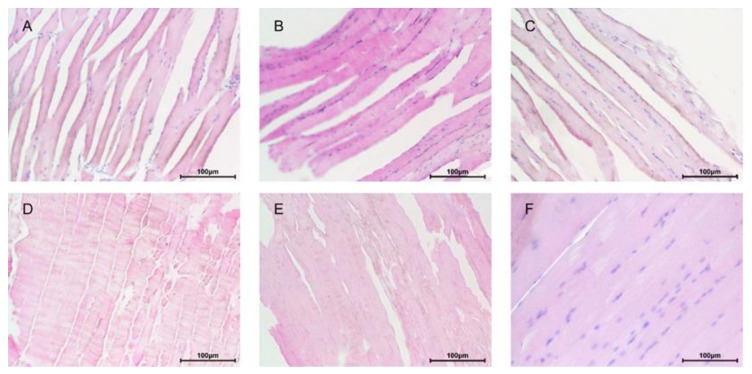
Muscle morphology was evaluated by H&E staining. (**A**) CTRL group; (**B**) Taurine group; (**C**) HPM-H group; (**D**) HPM-L group; (**E**) HPS-H group; (**F**) HPS-L group. Scale bar: 100 μm.

After the mice performed vigorous exercise, the skeletal muscle fibers of the CTRL group became larger and broken (Figure 2). The HPM and HPS groups had better muscle tissue morphology with less skeletal muscle fiber spacing and breakage. The HPS-L group showed most significant improvement, which had increased density and a more orderly arrangement of the muscle than the CTRL group (Figure 2).

### 3.4. Effects of the Spore and Mycelia of Paecilomyces hepiali on the Oxidative Stress and Glycogen Content

There was no significant difference in glutathione peroxidase (GSH), superoxide dismutase (SOD), and catalase (CAT), in mouse liver among groups, except a slight decrease of CAT was found in HPM-H group (Supplementary Figure S4A–C). Compared with the CTRL group, there is a significant reduction in the content of malondialdehyde (MDA) in the liver of HPM-H and HPS-L group. The content of MDA in the HPM-H group was reduced by 25%, and the HPS-L group was reduced by 39% (Figure 1G).

Although the level of liver glycogen in HPM and HPS groups was slightly higher compared with the CTRL group, there was no significant difference (Supplementary Figure S4D). The content of muscle glycogen in the TAU group was significantly higher compared with the CTRL group. However, there was no considerable difference between the CTRL and *P.Hepiali* groups (Figure S4E). These results indicate that the spore and mycelia of *P.Hepiali* may not regulate oxidative stress level or glycogen content to relieve fatigue.

### 3.5. Effects of the Spore and Mycelia of Paecilomyces hepiali on Gut Microbiota

To investigate the role of the spore and mycelia of *P.hepiali* on gut microbiota, we collected mouse fecal samples, extracted the DNA and performed high-throughput sequencing of 16S rRNA gene fragments. A total of 1524 ASVs were obtained, and the average count per sample was 40,522. The species abundance curve was established to estimate the adequacy of the sampling amount and the species richness. We found the upward trend at the end of the curve reached a plateau, proving that the sequencing result is credible (Supplementary Figure S5A). The rank abundance curve further verifies the adequacy of the sample size and the coverage of the species (Supplementary Figure S5B).

The Alpha diversity (α-diversity) indexes were used to evaluate the richness and diversity of the microbial communities. Although there were no significant changes between CTRL and all the five treatment groups, we found that the ACE, Chao1 and Shannon indexes decreased in TAU groups, while Simpson index increased in the TAU group (Supplementary Figure S6). Interestingly, compared with the TAU group, the ACE and Chao1 indexes significantly increased in the HPM-H group, the Shannon index increased in the HPS-L group, and the Simpson index decreased in the HPS groups (Supplementary Figure S6). These data indicate that *P.hepiali* promoted the richness and evenness of gut microbiota compared with taurine supplementation.

**Figure 3:**
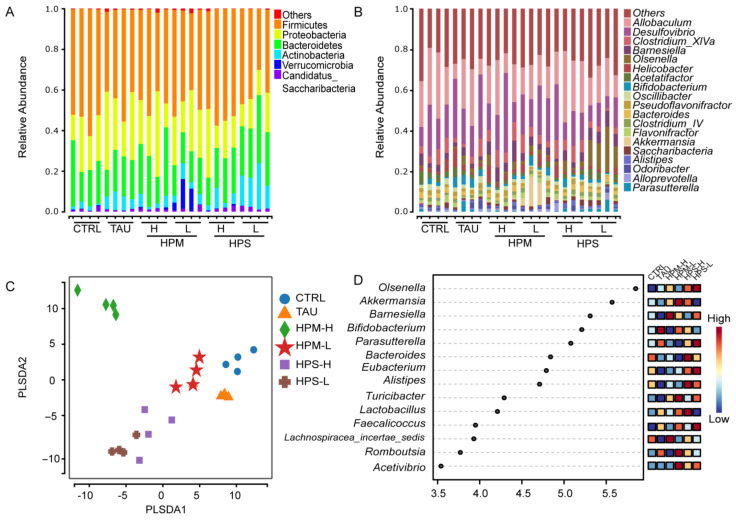
Effects of the spore and mycelia of *Paecilomyces hepiali* on the gut microbial composition. (**A**) Relative abundance of phyla; (**B**) Relative abundance of genera; (**C**) Partial least squares Discriminant Analysis, (PLS-DA); (**D**) Linear discriminant analysis Effect Size (LEfSe). The selected classification level was the genus level, the analysis method was Kruskal Wallis test, and the threshold of the log-linear discriminant analysis score was 2.0.

The gut microbial structure in all groups was analyzed at the level of phylum and genus classification. At the phylum level, Firmicutes, Proteobacteria, and Bacteroidetes were the dominant bacteria phyla. Compared with other groups, the relative abundance of Actinobacteria and Verrucomicrobia in HPS-L group mice was significantly higher (Figure 3A). At the genus level, the abundance of Acetatifactor in the HPM-H group was significantly higher (*p* < 0.05). The abundance of *Olsenella* in the HPS-L group was considerably higher, while the abundance of *Clostridium*_XlVa was significantly reduced (*p* < 0.05) (Figure 3A,B).

The dimensionality reduction analysis of the intestinal microbial structure was carried out to analyze the repeatability within the group and the difference between the groups. Partial least squares Discriminant Analysis (PLS-DA) showed separation between the different groups (Figure 3C). The HPS-H and HPS-L groups were closer, while the HPM-H group was farther away from the other groups. Points in each group was gathered but distinguished between different groups, indicating that difference in the dietary conditions successfully exerted a substantial effect on the structure of gut microbiota. The Beta diversity also differed significantly (Unweighted UniFrac; *p* < 0.05) (Supplementary Figure S7A). The 3D results of principal coordinate analysis (PCoA) further verified the separation between each group (Supplementary Figure S7B).

Then in order to explore the different microbial populations, determine the statistically significant characteristics, and evaluate the impact of the difference between groups, the output results were subjected to Linear discriminant analysis effect size analysis (LefSe) (Figure 3D and Supplementary Figure S8). HPS and HPM modulated the gut microbiota by increasing the abundance of *Akkermansia*, *Barnesiella*, *Bifidobacterium*, *Olsenella* and *Lactobacillus*. The relative abundance of *Alistips*, *Eubacterium*, *Bacterium*, *Parasutterella* and *Olsenella* in HPS group was higher than that in HPM group. *Olsenella*, an anaerobic Gram-positive bacterium, was enriched in all treatment groups. It can metabolize a series of monosaccharides and oligosaccharides to produce lactic acid. *Akkermansia* and *Lactobacillus* as gut probiotics were dominant in HPM-L and HPS-H groups. The results indicate that these bacteria could be identified as biomarkers.

The heatmap showed top 46 genera in the gut microbiota, and we found an up-regulation of the relative abundance of *Parasutterella*, *Bacteroides*, *Eubacterium*, and *Alistipes*, while a down-regulation of *Helicobactor* and *Escherichia* in HPS-H and HPS-L groups (Supplementary Figure S9). The HPM-L and HPM-H group showed an up-regulation of the relative abundance levels of *Romboutsia*, *Akkermansia*, *Bifidobacterium*, and *Lactobacillus*. These results indicate that different doses and parts of *P.hepiali* have different effects on the composition of the gut microbial community.

**Figure 4:**
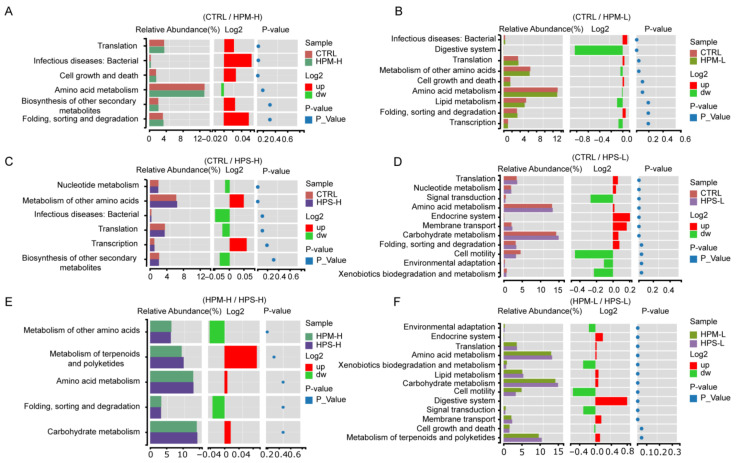
Function prediction of gut microbiota. On the left is the relative abundance histogram of each group; in the middle is the log2 value of the mean relative abundance ratio of the same pathway in the two groups; on the right is the *p*-value obtained by the Wilcox test. (**A**) CTRL Vs HPM-H; (**B**) CTRL Vs HPM-L; (**C**) CTRL Vs HPS-H; (**D**) CTRL Vs HPS-L; (**E**) HPM-H Vs HPS-H; (**F**) HPM-L Vs HPS-L.

**Figure 5:**
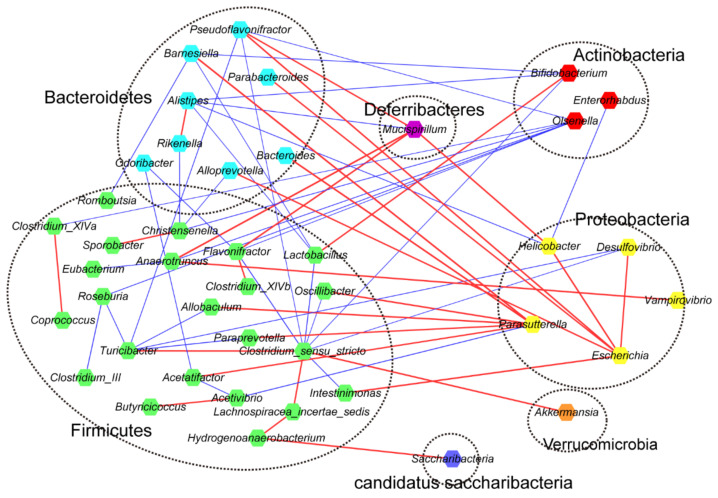
Correlation network generated using the SparCC algorithm. Each node in the Figure represents an ASV, the colors of the dots represent different microorganisms, and the microorganisms of the same phylum are in one area. The species are connected by straight lines. Red indicates positive correlation, blue indicates negative correlation, and the thickness of the line indicates the magnitude of the correlation.

Furthermore, we used KEGG function difference analysis to explore the potential functions of the gut microbiota of different groups. Compared with the CTRL group, functions related to nucleotide metabolism, metabolism of other amino acids capacity were increased in the HPS-H group, while functions related to translation, nucleotide metabolism, amino acid metabolism, endocrine system, membrane transport and carbohydrate capacity were increased in the HPS-L group (Figure 4). Correlation analysis showed a positive correlation between probiotic *Bifidobacteria* and *Lactobacilli*, which may promote each other’s growth in the intestinal tract and regulate intestinal balance. *Akkermansia*, a probiotic that can improve host metabolism, was positively correlated with *Clostridium sensu stricto*. The genera in Proteobacteria were mainly positively correlated with genera in other phyla, whereas the genera in Actinomycetes were negatively correlated with most of genera in other phyla (Figure 5).

### 3.6. Correlation between Gut Microbiota and Anti-Fatigue Metabolites

**Figure 6:**
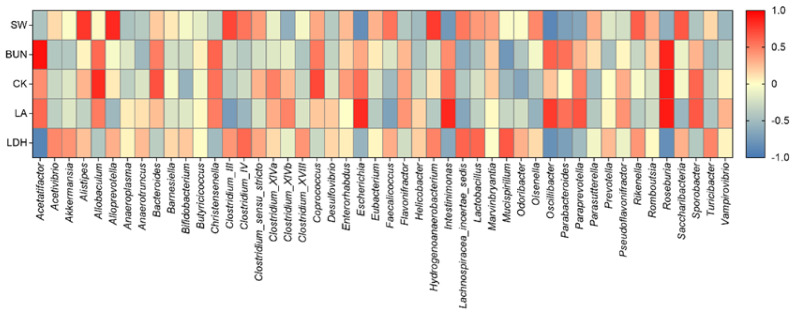
The correlation analysis between gut microbiota and anti-fatigue physiological characteristics. The correlation coefficient ranged from −1.0 to 1.0. Red and blue indicate positive correlation and negative correlation, respectively. The SW indicated the exhaustive swimming time, LD represented lactic acid, BUN represented blood urea nitrogen, CK represented creative kinase, LDH represented lactate dehydrogenase.

To explore the correlation between gut microbiota and anti-fatigue physiological characteristics, we used Spearman correlation analysis. The SW and LDH content were positively correlated with *Alistips*, *Clostridium*, *Akkermansia*, *Olsenella*, and *Lactobacillus*, while the BUN, CK, and LA levels were significantly and positively correlated with the abundances of *Acetatifactor*, *Allobaculum*, *Oscillibacter*, *Paraprevotella*, and *Roseburia* (Figure 6).

## 4. Discussion

As a sub-healthy physiological state, fatigue is a precursor to many diseases [24]. In this study, we studied the chemical composition and anti-fatigue effect of HPS and HPM. We found that 85 metabolites were significantly differentially abundant in HPS and HPM by LC-MS/MS analysis. The HPS contain more Fructose-1,6-bisphosphate, D-Glucuronic acid, malic acid, and oxalacetic acid, which can provide the energy sources. HPS significantly prolonged the forced swimming time.

*P.hepiali* has been studied for its toxicicity, and it has been proven to be non-toxic to humans [15]. As reported, there is no significant difference in the body weight and main organ indexes of the mice in our study. LDH and LA in the blood are important indicators for measuring fatigue [25]. BUN is the main component of serum non-protein nitrogen and is considered to be the main end product of human protein metabolism [26]. CK is an important kinase that is directly related to intracellular energy transport, muscle contraction, and ATP regeneration. Exercise can cause a significant increase in CK [27]. Decrease of CK level in mice indicates less accumulation of metabolites during exercise, and therefore lower degree of fatigue. In the present study, we found that the levels of LA, BUN and CK were down-regulated, while the level of LDH was up-regulated in both the HPM and HPS groups compared with the blank control group. However, indicators of other anti-fatigue ability, such as active oxygen free radicals, liver glycogen, and muscle glycogen, have no significant difference, indicating that the anti-fatigue ability of *P.hepiali* may not be achieved by the accumulation of glycogen or the regulation of oxidative stress.

Taking account of the ability of *P.hepiali* as a prebiotic and the relationship between the gut microbiota and body fatigue, we inferred that *P.hepiali* can act as a prebiotic to regulate the structure of the gut microbiota of mice [28]. We found that the gut microbial α diversity of the *P.hepiali* groups has increased compared with that of the positive TAU group. Regardless of the dosage and whether it is mycelia or spores, *P.hepiali* can modify the microbial composition in the intestinal tract.

The relative abundance of *Alistips* in HPS groups was significantly up-regulated compared with HPM groups and other groups. *Alistips* is a genus of anaerobic bacteria found mostly in healthy human gut microbiota [29]. There was a correlation of the relative abundance of *Alistips* in gut microbiota with people suffering from chronic fatigue syndrome [30]. However, the causal relationship between *Alistips* and fatigue is still unknown. Future studies should be conducted to verify the role of *Alistips* in fatigue.

Other than *Alistips*, we also found that *Clostridium*, *Akkermansia*, *Olsenella*, and *Lactobacillus* were positively correlated with the SW and LDH content by correlation analysis. These could be potentially beneficial bacterial strains. *Lactobacillus spp.* are important gut beneficial microbes, which can maintain the balance of gut microbiota, enhance immunity, provide essential nutrients for the host, enhance nutritional metabolism, and promote the growth of the body [31]. The bile saline hydrolase (BSH) possessed by *Lactobacillus* can also deconjugate the conjugated bile acid [32]. The storage of bile acids can cause muscle fatigue [33]. The production of organic acids is an important way for gut microbiota to regulate the human host [34]. Short chain fatty acids produced by gut microbiota have special physiological functions [35]. *Akkermansia* can use mucin as the sole source of carbon and nitrogen for energy metabolism [36,37]. It is also closely related to the body’s digestion and metabolism, can improve the metabolic status, and is considered a new type of probiotics. It exhibits a series of potential beneficial effects on the health of the host, and is believed to treat obesity, diabetes, and inflammatory bowel disease [38]. It can promote the production of butyrate by cross-feeding [39]. This provides gut health benefits to the host [40]. Further study is needed to understand how these microorganisms participate in the anti-fatigue effect of *P.Hepiali*.

## 5. Conclusions

In conclusion, our study illustrates that *P.hepiali* spores, as a supplementary functional food, exhibit significant anti-fatigue effect by modulating gut microbiota in mice.

## Data Availability

The data are publicly available online (https://nmdc.cn/, accessed on 7 December 2021). BioProject: NMDC10017933. The datasets used and analyzed during the current study are available from the corresponding author upon reasonable request.

## Supplementary Materials

The following supporting information can be downloaded at: https://www.mdpi.com/article/10.3390/nu14142973/s1, Figure S1: Contents of general components of mycelium and spores of *Paecilomyces hepiali*.; Figure S2: Heatmap comparison based on the 85 metabolites with significant differences.; Figure S3: The body weight changes of mice on 0 day and 28 day.; Figure S4: The anti-fatigue effect of the HP on mice in each group, (A) Glutathione peroxidase (GSH), (B) Superoxide dismutase (SOD), (C) Catalase (CAT), (D) Hepatic glycogen (HGN), (E) Muscle glycogen (MGN); Figure S5: Evaluation of sequencing data of the different bacterial community groups. (A) ASVs accumulation curve, (B) Rank abundance curve.; Figure S6: Alpha diversity of the gut microbiota. (A) ACE index, (B) Chao 1 index, (C) Shannon index, (D) Simpson index.; Figure S7: Beta diversity of the gut microbiota. (A) The intergroup differences of Beta diversity of the mice gut microbiota, (B)Principal Co-ordinates Analysis (PCoA) of the mice gut microbiota based on Bray–Curtis metrics; Figure S8: Linear discriminant analysis (LDA) scores derived from LEfSe analysis. (A) CTRL Vs TAU, (B) CTRL Vs HPM-H, (C) CTRL Vs HPM-L, (D) CTRL Vs HPS-L, (E) CTRL Vs HPS-H.; Figure S9: Heatmap comparison based on the top relative abundance of 46 key genus.; Table S1: Body and organ weight of mice.
