# Supplementary material for: Spore Powder of Paecilomyces hepiali Shapes Gut Microbiota to Relieve Exercise-Induced Fatigue in Mice
Source: Nutrients. 2022 Jul 20;14(14):2973. doi: 10.3390/nu14142973 (PMC9323605; doi:10.3390/nu14142973)
Supplement: Supplementary file 1 [file nutrients-14-02973-s001.zip › nutrients-1819069-supplementary.pdf]

Supporting Information

Table S1. Body and organ weight of mice.

group	Weight (g)	Organ index (%)				
		heart	liver	spleen	lung	kidney
Control	23.74±1.67	0.53±0.08	3.51±0.18	0.24±0.03	0.59±0.05	1.11±0.33
TAU	24.70±1.54	0.48±0.05	3.37±0.15	0.26±0.04	0.59±0.08	1.19±0.10
PHM-H	24.47±1.53	0.52±0.05	3.38±0.24	0.26±0.04	0.60±0.07	1.23±0.05
PHM-L	22.91±1.41	0.58±0.08	3.44±0.16	0.26±0.03	0.63±0.09	1.26±0.07
PHS-H	23.66±1.27	0.55±0.05	3.48±0.08	0.23±0.03	0.59±0.06	1.29±0.07
PHS-L	24.51±1.13	0.52±0.08	3.43±0.12	0.25±0.03	0.59±0.06	1.27±0.08

Values are expressed as means ± SD (n = 10).

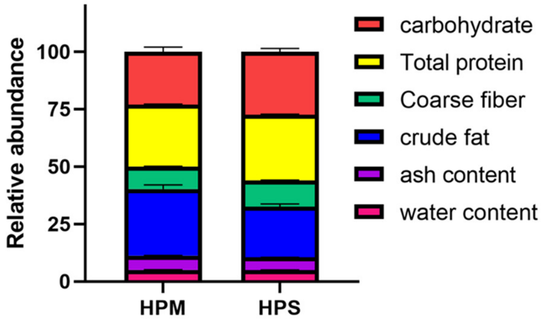

Figure S1. Contents of general components of mycelium and spores of *Paecilomyces hepiali*.

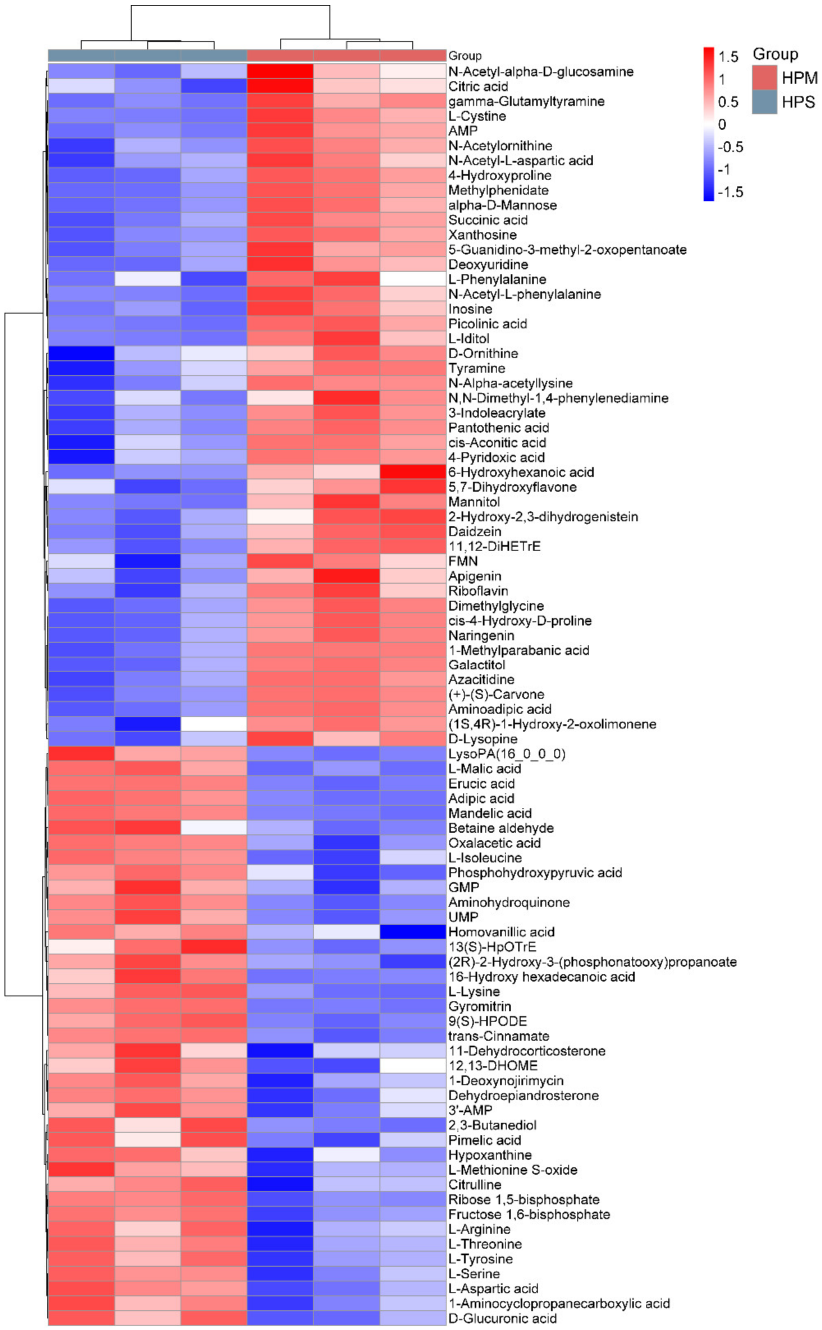

Figure S2. Heatmap comparison based on the 85 metabolites with significant differences. The intensity of the color of the heatmap represents the degree of relative abundance.

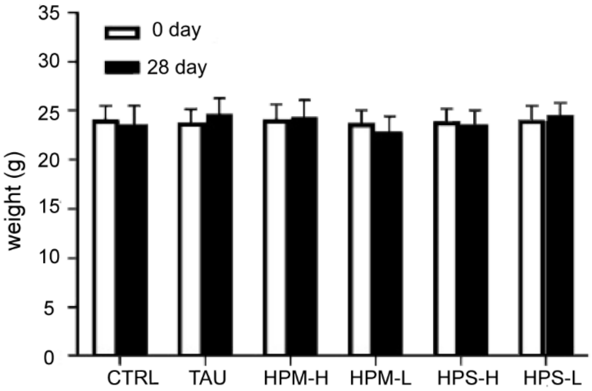

Figure S3. The body weight changes of mice on 0 day and 28 day. Data are mean ± SD (n = 10 per group).

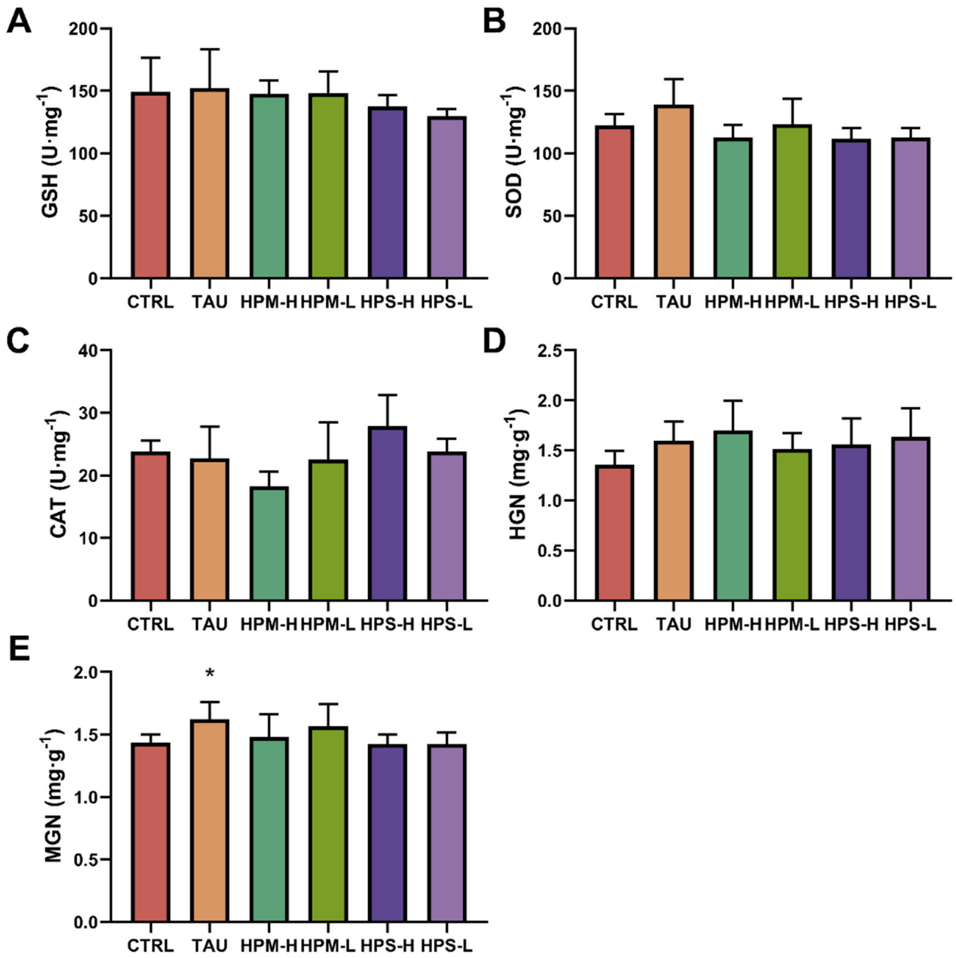

Figure S4. The anti-fatigue effect of the HP on mice in each group. (A) Glutathione peroxidase (GSH); (B) Superoxide dismutase (SOD); (C) Catalase (CAT); (D) Hepatic glycogen (HGN); (E) Muscle glycogen (MGN).

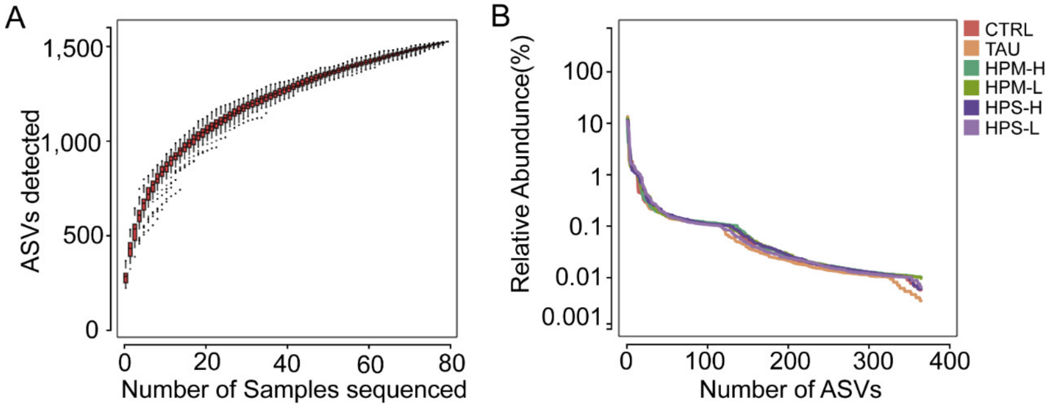

Figure S5. Evaluation of sequencing data of the different bacterial community groups. (A) ASVs accumulation curve; (B) Rank abundance curve.

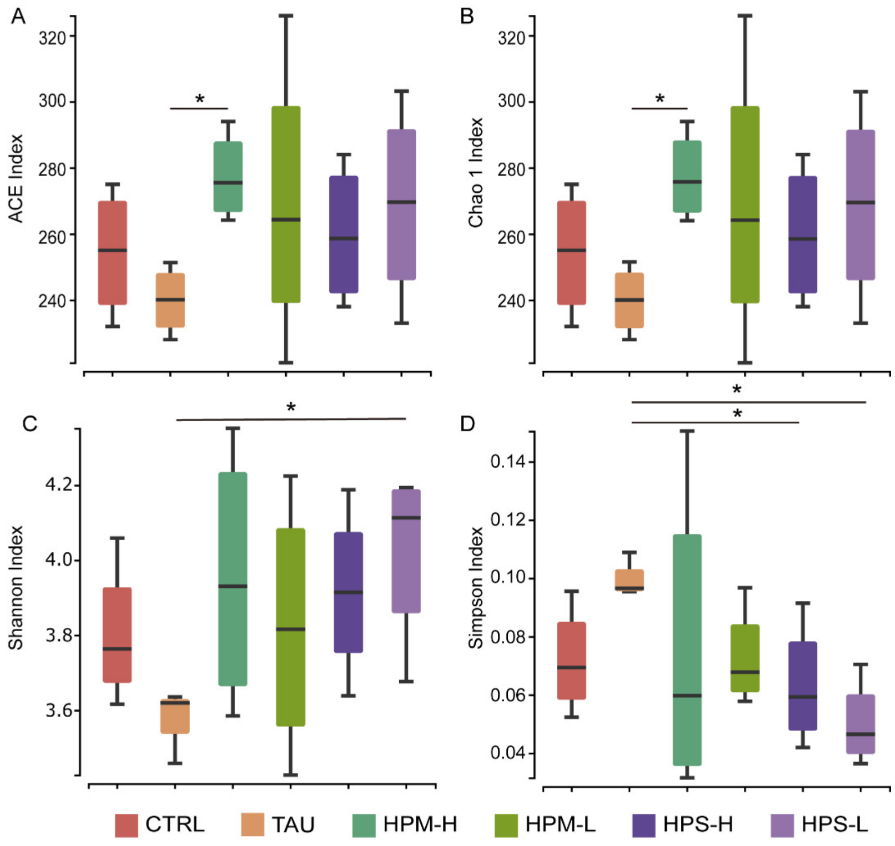

Figure S6. Alpha diversity of the gut microbiota. (A) ACE index; (B) Chao 1 index; (C) Shannon index; (D) Simpson index. Asterisk (*) represents $p < 0.05$.

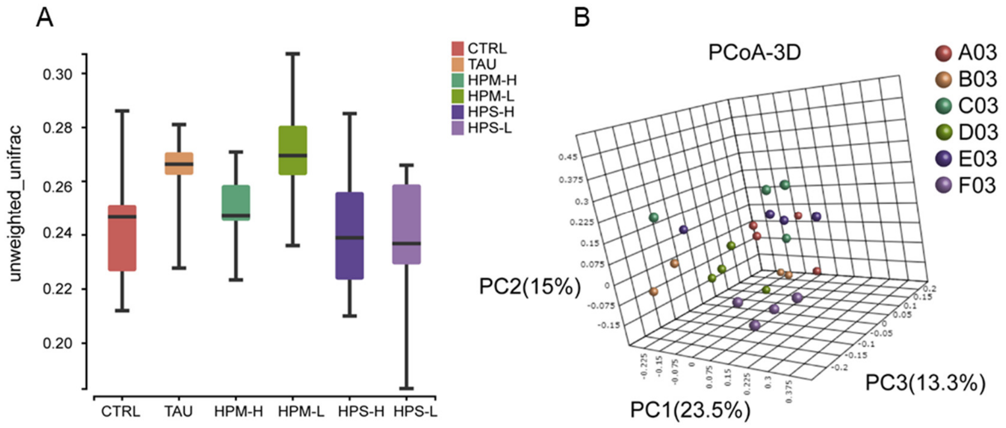

Figure S7. Beta diversity of the gut microbiota. (A)The intergroup differences of Beta diversity of the mice gut microbiota; (B) Principal Co-ordinates Analysis (PCoA) of the mice gut microbiota based on Bray–Curtis metrics.

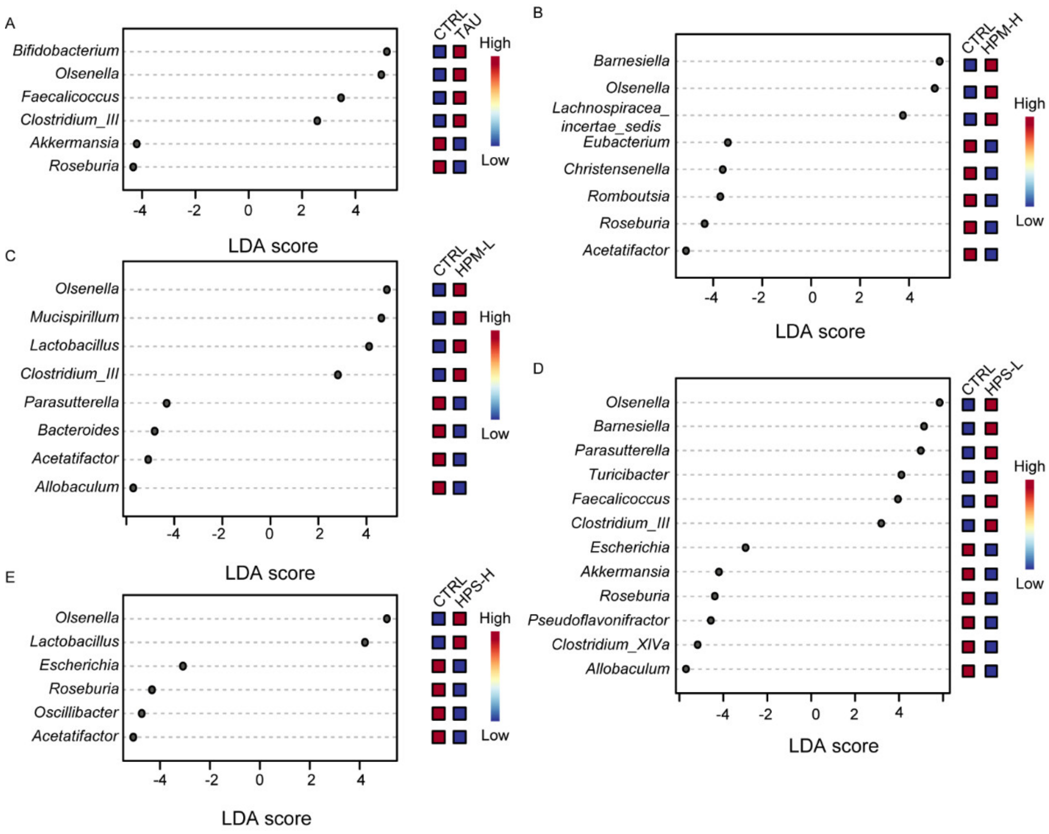

Figure S8. Linear discriminant analysis (LDA) scores derived from LEfSe analysis. The biomarker taxa LDA score of > 2 was shown (the length of the bar represents the LDA score). (**A**) CTRL Vs TAU; (**B**) CTRL Vs HPM-H; (**C**) CTRL Vs HPM-L; (**D**) CTRL Vs HPS-L; (**E**) CTRL Vs HPS-H.

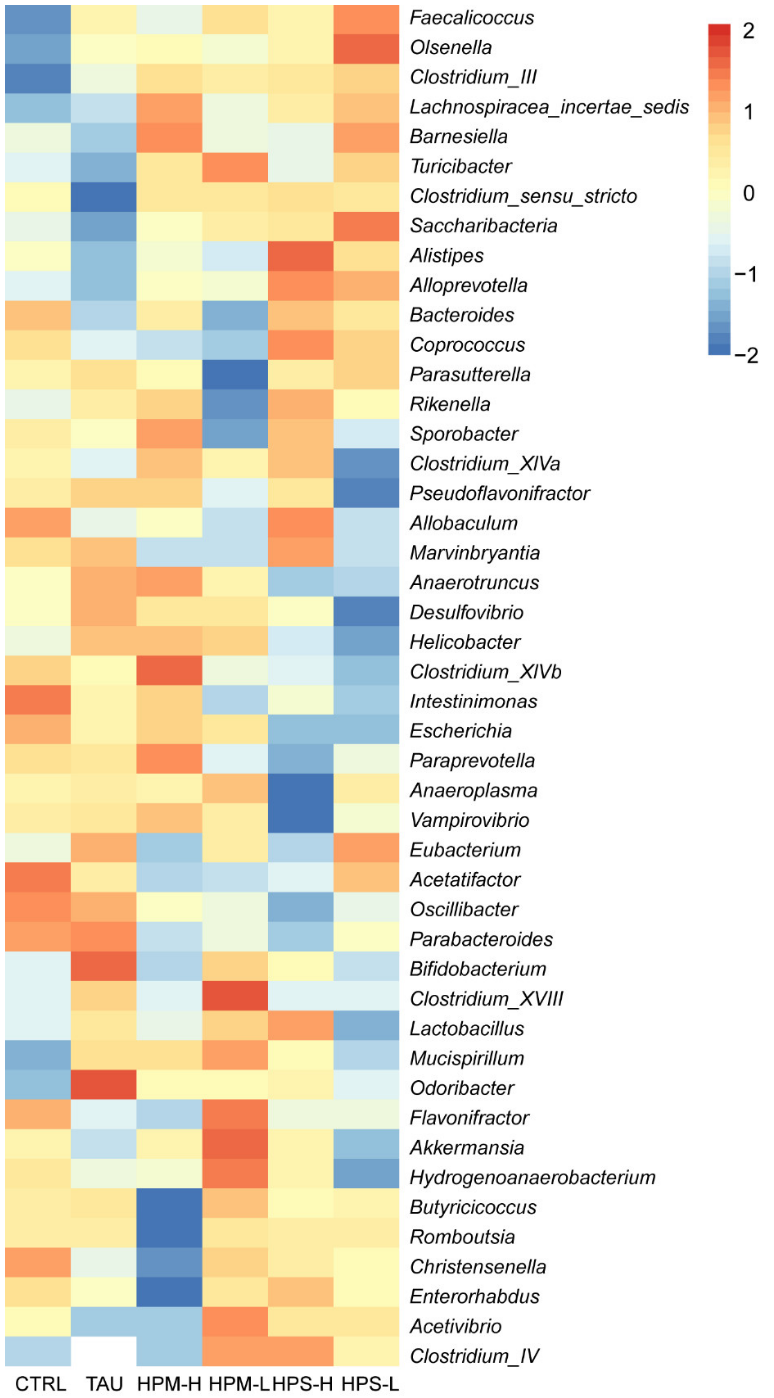

Figure S9. Heatmap comparison based on the top relative abundance of 46 key genus. The intensity of the color of the heatmap represents the degree of relative abundance.
